# Multi-objective sustainable supply chain network optimization based on chaotic particle—Ant colony algorithm

**DOI:** 10.1371/journal.pone.0278814

**Published:** 2023-07-10

**Authors:** Tianrui Zhang, Wei Xie, Mingqi Wei, Xie Xie

**Affiliations:** 1 School of Mechanical Engineering, Shenyang University, Shen Yang, China; 2 School of Control Science and Engineering, Dalian University of Technology, Dalian, China; 3 Key Laboratory of Manufacturing Industrial and Integrated Automation, Shenyang, China; Guru Ghasidas Vishwavidyalaya: Guru Ghasidas University, INDIA

## Abstract

For the optimal design of the sustainable supply chain network, considering the comprehensiveness of the problem factors, considering the three aspects of economy, environment and society, the goal is to minimize the establishment cost, minimize the emission of environ-mental pollution and maximize the number of labor. A mixed integer programming model is established to maximize the efficiency of the supply chain network. The innovation of this paper, first, is to consider the impact of economic, environmental and social benefits in a continuous supply chain, where the environmental benefits not only consider carbon emissions but also include the emissions of plant wastewater, waste and solid waste as influencing factors. Second, a multi-objective fuzzy affiliation function is constructed to measure the quality of the model solution in terms of the overall satisfaction value. Finally, the chaotic particle ant colony algorithm is proposed, and the problem of premature convergence in the operation of the particle swarm algorithm is solved. Experimental results show that the PSCACO algorithm proposed in this paper is compared with MOPSO, CACO and NSGA-II algorithms, and the convergence effect of the algorithm is concluded to be more effective to verify the effectiveness and feasibility of chaotic particle ant colony algorithm for solving multi-objective functions, which proposes a new feasible solution for the supply chain management.

## Introduction

Nowadays, with the rapid development of global industrialization, the manufacturing industry and supply chain networks are becoming significantly concerned about sustainability indicators [1], while in order to achieve sustainable development, the industry needs to re-plan the original supply chain [2]. As one of the important responsible subjects of resource consump-tion, environmental pollution and promotion of social environmental awareness, the design and optimization of sustainable supply chain network has become the focus of attention of all countries in the world. In addition, the balanced impact of business on total cost, environ-mental and social responsibility has become a continuing issue. The impact of supply chain network design on business effectiveness is becoming more prominent, and the public and government are very concerned about the sustainability of supply chain companies. In recent years, due to the continuous improvement of environmental regulations and awareness, and also for the purpose of reducing pollution and alleviating customer pressure, issues such as reverse logistics, green manufacturing and remanufacturing, waste management and reuse are considered as green supply chain management. The important content in Chain Management is increasingly attracting the attention of the academic and business circles. Lin et al. [3] divided the solutions to the closed loop green supply chain network design problem into the following three categories: optimizing fuel consumption, reducing exhaust gas and carbon dioxide emissions, and managing waste and used items. Li Jin et al. [4] aimed at the strategic positioning and configuration of multi-level closed-loop supply chain network design under low-carbon environment, considered the ambiguity of supply chain network parameters and the problem of multi-product flow, and considered the total cost and carbon emissions of supply chain network. The minimum is the objective, and a multi-objective robust fuzzy op-timization model is established. As for the exhaust gas and carbon emissions that lead to en-vironmental problems, Lamba et al. [5] discussed the problem of supplier selection in a low-carbon environment, and modeled carbon emission cost control as one of the criteria for supplier selection.

Due to the emphasis on the environment and society, supply chain network design issues are more complex than in the past [6], but at the same time, implementing sustainable supply chain management can minimize the negative environmental impact of the supply chain, en-hance public acceptance, and bring the company into a virtuous cycle while enhancing bene-fits for the company. Therefore, it is particularly important to study the construction of a balanced and efficient sustainable supply chain network planning model.

Traditional supply chain network design management has failed to address environmental and social issues [7], such as CO_2_ pollution from transportation processes, control of carbon emissions in the production environment [8]. Today, with the increasing environmental awareness, scholars have proposed the concept of closed-loop supply chain management, considering the recycling and remanufacturing concepts and introducing carbon emissions into traditional supply chain management [9]. Business operations managers need to consider economic and social influences in supply chain networks, most studies have considered economic aspects, Fathollahi-Fard et al. [10] consider both economic and social aspects to study multi-objective stochastic closed-loop supply chain design.

In theory, in the study of predecessors for supply chain network design, the establishment of the objective function of mainly includes the economic and environmental benefits, this article explains the social responsibility after the positive influence to sustainable development, combined with stakeholder theory and the selection criteria in this paper, based on the objective function of the social responsibility. Secondly, in terms of modeling, the sustainable development at the macro level is extended to the sustainable development of supply chain at the micro level. Social responsibility index is added to the objective function, social benefit objective function is established, its overall performance optimal problem is studied, and the content, theory and method of traditional supply chain network design are expanded.

Based on this, in view of the multi-level sustainable supply chain network problem, this paper considers the three aspects of economy, environment and society, and considers carbon emission and environmental pollution in environmental benefits, which are rarely covered in previous research literature. After that, the amount of labor created by the network for the society is used as an indicator of social benefit. Moreover, this paper constructs a multi-objective mixed integer programming model with the goals of minimizing network oper-ating costs, carbon and pollution emissions, and maximizing labor. For the processing of the model, based on the fuzzy affiliation theory, the affiliation function of each objective function is given, and the quality of the model solution is measured using the maximization satisfaction index, while a chaotic particle ant colony algorithm solution model is proposed. In the algorithm design, in the multi-objective programming problems, such as the solution of two objective functions, many experts use ant colony algorithm, particle swarm optimization algorithm and genetic algorithm, the operation process of the algorithm is relatively tedious; In this paper, three objective functions are established. If the above methods are adopted, the solution process will be more complicated. Therefore, in this paper, the membership function of three objective functions is established, the multi-objective optimization problem is converted into a single objective value, and the ant colony algorithm is used to improve the search speed to improve the particle swarm optimization algorithm to make the solution more simple, and ensure that the final solution is the Pareto optimal solution. Finally, the feasibility of the constructed model and the efficiency of the proposed algorithm are verified by example simulation.

## Literature review

Sustainable supply chain network design (SSCND) is a top-level strategic decision for supply chain networks. In recent years, academics have closely aligned supply chain network design with the 2030 Agenda for Sustainable Development strategy released by the United Nations to achieve balanced economic, environmental and social development [11]. While the design of the supply chain is a complex task, the problem is solved by building a mixed integer linear model [12].

A large number of scholars are currently conducting research, Yadav et al. [13] omnichannel environmental sustainable supply chain network design, investigating bi-objective optimiza-tion to reduce carbon content as well as minimize supply chain costs, and developing a mixed-integer linear programming model. Zhen et al. [14] proposed to develop a green and sustainable closed-loop supply chain network under uncertain demand by developing a bi-objective optimization model of CO_2_ emissions and total operating costs, considering both environmental and economic issues. Diabat et al. [15] considered the closed-loop supply chain network design problem for recycling durable products, and proposed a multi-product, multi-cycle mixed integer linear programming model by considering both environmental and economic approaches. Moheb-Alizadeh et al. [16] developed a stochastic integrated mul-ti-objective mixed integer nonlinear programming model to solve the efficiency problem in a sustainable closed-loop supply chain network, considering environmental and social impacts. Eskandarpour et al. [17] studied the sustainable supply chain network design problem and proposed a bi-objective mixed integer planning model that addresses minimizing logistics costs as well as CO_2_ emissions (raw material supply manufacturing, warehousing and transportation) as an objective. Yavari et al. [18], in their study of the green and resilient supply chain network disruption risk problem, the risk mitigation strategy considers the economic and environmental impacts and develops a bi-objective mixed integer linear programming model.

As far as the above literature is concerned, most of the current literature has conducted mod-eling optimization studies on both economic and environmental aspects, respectively. The existing literature on sustainable supply chain networks by previous authors has adopted an operations research perspective to address the issues of reducing carbon emissions and min-imizing supply chain costs, however, the social issues have not been well addressed to a large extent. As a result, some scholars have studied sustainable supply chains to include social influences. Nayeri et al. [6] study the economic, environmental and social impact optimiza-tion objective model for sustainable closed-loop supply chains to configure the uncertainty present in the supply chain network problem according to the changes in the business envi-ronment (e.g., transportation costs and demand). Shabbir et al. [19] proposed a closed-loop supply chain design based on uncertainty and sustainability, using a two-stage stochastic planning approach that considers environmental, social and total operational costs. Jouzdani et al. [20] developed a multi-objective mathematical planning model to optimize opera-tion-related costs, energy consumption and traffic congestion in supply chains, considering three aspects social, environmental and economic, all to improve the sustainability of supply chain network design. Lahri et al. [21] proposed a two-stage multi-objective integer linear programming sustainable supply chain network design model with the objective of mini-mizing economic, environmental and maximizing social sustainability.

According to Table 1, the current research on the supply chain needs to consider cost, energy and personnel resource allocation. Therefore, many factors can be considered in the design of a sustainable supply chain network, such as the economic aspect to consider equipment re-manufacturing, the environmental aspect to consider carbon neutrality, and the social aspect to consider policy and social employment needs. At the same time, there is still a lack of literature research that considers the three goals of economy, environment and society at the same time. Therefore, it is meaningful to consider the design of supply chain network from these three goals. For the objective function, most scholars choose to establish a multi-objective mixed integer programming model.

**Table 1:** Literature summary.

Literature	Considering	Target			Objective Function	Sustainability
		Economy	Environment	Society	Mixed Integer Programming Model	
12	Carbon Content, Total Cost	√	√		√	√
13	CO2\Total Operating Cost	√	√		√	√
14	Recycled Products	√	√		√	Closed-loop supply chain network
15	Network efficiency		√	√	√	√
16	Logistics costs\CO2	√	√		√	√
17	Profit	√		√	√	√
18	Network outage risk	√	√		√	Green and resilient supply chain network
19	Business Environment Changes	√	√	√	√	√
20	Cost, Environment and People	√	√	√	√	√
21	Operational related costs, energy consumption and traffic congestion	√	√	√	Mathematical programming model	√
22	Minimize Cost, Environment, Maximize Society	√	√	√	√	√

Meanwhile, for the multi-objective function solution problem in sustainable supply chain networks, a mixed integer linear programming (MILP) optimization is generally established, which is a typical NP-Hard problem [22]. With the increase of problem size and system com-plexity, traditional exact algorithms are not applicable and heuristic algorithms are needed to solve them. Particle Swarm Optimizer (PSO) [23] has been widely used for solving NP-hard problems because of its fast convergence, few parameters and simple operation. Che [24] proposed an improved PSO algorithm for solving the mathematical model established for the multi-echelon unbalanced supply chain planning problem. Zhao et al. [25] proposed a mul-ti-objective improved particle swarm optimization algorithm to find non-dominated solutions to optimize the four-echelon supply chain network design combined with transportation mode selection. Surco et al. [26] used a particle swarm optimized water distribution network model that considers both real and discrete variables and uses an objective function to avoid premature convergence to a local optimum. Tabibi [27] proposed an algorithm based on par-ticle swarm optimal selection to select the best intersection point for wireless sensing networks to achieve efficient management of network resources. The drawback of the particle swarm algorithm is that the search is prone to premature in the later stages, so the algorithm needs to adopt a method to help it jump out of the local optimum in the later stages. The disadvantage of the particle swarm algorithm is that it tends to be premature in the later stages of the search, so the algorithm needs to adopt a method to help it jump out of the local optimum in the later stages.

For supply chain problem, Chaotic initial population generation PSO with levy flight dis-tribution has been applied for solving the mathematical model for this multi-channel closed loop supply chain by taking into consideration the economic and environmental objec-tives [28]. A particle swarm optimization algorithm based on Pareto archive that utilizes the genetic algorithm operators is proposed to solve the mixed integer programming model is proposed in which the profit maximization and the CO_2_ emission minimization are formu-lated [29]. A cooperative game model of supply chain logistics information based on collab-orative immune quantum particle swarm optimization is proposed. The Nash equilibrium solution in the cooperative game model of supply chain logistics information is taken as the optimization particle, the global optimal solution of the cooperative game model of supply chain logistics information is obtained [30]. Dual Fitness PSO (DFPSO) is proposed that not only fitness and diversity of the particles are properly evaluated, but also the abilities to evaluate these features are integrated to avoid the above-mentioned problem in determining the global guide particles. For the improvement of particle swarm optimization, most scholars choose to improve the speed of particle update [31]. The improved particle swarm algorithm can make the result better than the algorithm without improvement, so that the algorithm can avoid falling into local optimum. Therefore, this paper uses the ant colony algorithm to improve the particle swarm optimization algorithm to improve the defects of this algorithm.

Kong et al. [32] proposed that the dominant and non-dominant solutions of the algorithm use various learning strategies to enable targeted learning and evolution of the particle swarm algorithm. Sedak et al. [33] improved the variational algorithm of the differential evolutionary algorithm to the velocity update equation of the particle swarm algorithm and used the adap-tive population spacing parameter to select the appropriate variational operator, for which a hybrid particle swarm differential evolutionary algorithm was proposed to solve the mul-ti-objective optimization. Ant Colony Optimization (ACO) has good robustness and is not easy to fall into local optima [34–36]. Therefore, the combination of particle swarm Optimi-zation and Ant Colony Optimization can effectively escape from local optima. Soleimani et al. [37] proposed a hybrid particle swarm algorithm and genetic algorithm to solve the problem based on the advantages and disadvantages of particle swarm algorithm and genetic algorithm when studying the problem of designing a large-scale network closed-loop supply chain network. Lu et al. [38] combined the global search capability of PSO with the powerful evolutionary capability of ACO and a bit of positive feedback to solve the multi-objective supply chain partner integration problem.

In recent years, scholars have applied particle swarm algorithm and ant colony algorithm to jointly solve different problems. Chen [39] proposed a multigroup ant colony optimization (MACO) algorithm that was used to determine the shortest trajectory with obstacle avoidance. A quantum-behaved particle swarm optimization (QPSO) algorithm was subsequently used to determine the optimal positioning error for each moving point along the shortest trajectory. Piskin [40] use a hybrid optimization code as a new approach. It can be used with other engine functions; for instance functions corresponding to turbo-shaft or turbofan engines, by modifying the engine function. Number of input parameters and objective functions can be modified accordingly. Aiming [40] at vehicle routing problem and combining the advantages of ant colony and particle swarm optimization, an intelligent optimization algorithm of adaptive ant colony and particle swarm optimization is proposed.

In addition, considering that there is no significant difference in pheromone concentration of each path in the initial stage of ant colony algorithm, the blindness of ant colony search will increase and the initial convergence speed will weaken.

To sum up, at present, in terms of sustainable closed-loop supply chain network design models to build mixed integer planning models, most studies only consider economic benefits or target both economic and environmental benefits, and there is less literature on modeling that considers three aspects: economic, environmental and social. Meanwhile, for the mul-ti-objective sustainable closed-loop supply chain network planning problem, which is an NP-hard problem, the existing algorithms are still inadequate to meet the practical needs. Therefore, it is important to construct a realistic model for sustainable supply chains that takes into account the three benefits, and to propose an algorithm that can be solved quickly and efficiently.

## Sustainable supply chain model construction

### Problem description

**Fig 1:**
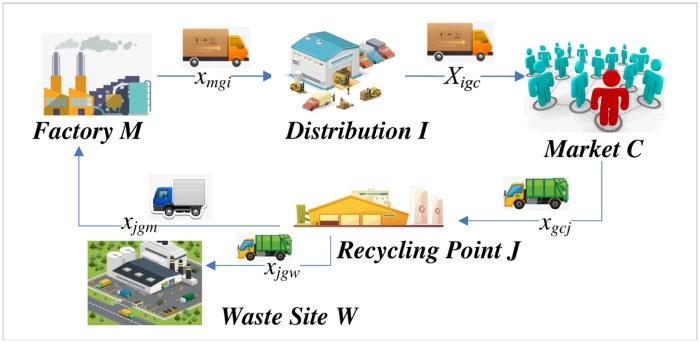
Network structure of sustainable closed loop supply chain.

The sustainable supply chain network constructed in this paper is shown in Fig 1. Fig 1 illustrates the sustainable supply chain network in this paper, including the plant, the distribution, market, recycling waste, and the plant through the introduction of different techniques to produce the product, the distribution is responsible for the products of the factory distribution to each market, collection points for consumer products has been used for recycling, and parts of products available for renovation. After that, the refurbished parts will be transported to the factory for recycling in production, while the products with no use value will be transported to the waste site for scrapping.

The assumptions of this article are as follows:

1. Factory, distribution point, recovery point and waste point all have capacity limits, and forward logistics and reverse logistics have the same capacity ratio;
2. The location of the plant is fixed, and the locations of alternative distribution points, recycling points and waste points are known;
3. Inventory at the factory, distribution point and recovery point is not considered;
4. The plant produces a fixed range of products;
5. Plants do not have the same exhaust emissions, wastewater emissions, and solids emis-sions when products are manufactured;
6. Possible product recovery and refurbishment rates are known;
7. Only the same means of transport can be used between each node, and the capacity of the means of transport is limited;
8. Refurbished parts for the period will be the source of the re-manufactured products for the period.

### Notations in the model

#### The meaning of notations

Each department and node of the supply chain network in this paper has a corresponding symbol. This section is a brief explanation of the subscript notation, parameter notation, and parameter notation related to the model of supply chain networks. Tables 2 and 3 are as follows.

**Table 2:** Representation of the subscript notation.

Notations	Meaning
*M*	stands for the factory number, *M* = {1, 2, …, *m*}
*I*	Distributor Number, *I* = {1, 2, …, *i*}
*J*	recycling center number, *J* = {1, 2, …, *j*}
*W*	represents the number of waste points, *W* = {1, 2, …, *w*}
*C*	represents the consumer market number, *C* = {1, 2, …, *c*}
*T*	stands for the manufacturing technology number, *T* = {1, 2, …, *t*}
*G*	Transport number, *g* = {1, 2, .., *g*}

**Table 3:** Representation of parameter symbol.

Parameter symbol	Meaning
*FC*_*e*_	Fixed cost of facility *e* opening, *e* = {*i*, *j*, *w*}
*IC*_*mt*_	The cost of introducing technology *t* to plant *m*
*PC*_*mt*_	Plant m uses technique *t* to generate the unit manufacturing cost of the product
*SC*_*m*_	Cost savings from remanufacturing of refurbished parts in plant *m*
*TC*_*g*_	Unit transport cost of transporting the product by means of transport *g*
*RC*_*j*_	Recovery point *j* The unit recovery cost of the recovered product
*NC*_*j*_	Unit refurbishment cost of refurbished product at recovery point *j*
*DC*_*w*_	Unit waste cost of waste disposal products at waste point *w*
*GC*_*g*_	Leasing cost of conveyance *g*
*EC*_*e*_	Fixed carbon emissions for facility e opening, *e* = {*i*, *j*, *w*}
*IEC*_*mt*_	The carbon emissions of plant m by introducing technology *t*
*PEC*_*mt*_	Plant m uses technology *t* to produce the unit carbon emissions of the products
*TEC*_*g*_	Carbon emissions per unit of product transported by means of transport *g*
*NEC*_*j*_	Carbon emissions per unit of the refurbished product at recycling point *j*
*DEC*_*w*_	Unit carbon emission of waste disposal products at waste point *w*
*DEP*_*mn*_	Waste gas emissions produced by plant m producing unit of product *n*, *N* = (1, 2, …, *n*)
*WEP*_*mn*_	Wastewater discharge from plant m producing unit product *n*
*SEP*_*mn*_	Plant m produces solid waste emissions per unit of output of product *n*
*L*_*e*_	The amount of labor required to open Facility *e*, *e* = {*i*, *j*, *w*}
*IL*_*mt*_	The carbon emissions of plant M by introducing technology *t*
*GL*_*g*_	The amount of labor required to maintain vehicle *g*
*QL*_*t*_	The employment coefficient of the product produced by technology *t*
*DIS*_*ab*_	The distance between node a and node *b*, *a* = {*m*, *i*, *c*, *j*}, *b* = {*i*, *c*, *j*, *w*, *m*}
*R*_*c*_	The product recovery rate of market *c*
*A*_*j*_	Refurbishment rate of product parts at recovery point *j*
*CA*_*m*_	The production ceiling of factory *m*
*CA*_*g*_	Maximum loading capacity of vehicle *g*
*CA*_*e*_	The upper limit of the product to be operated in Facility *e*

#### Decision variables

Binary decision variable

$$\begin{array}{c} OP_{e}=\{\begin{array}{c} 1,\;\text{Opening}\;\text{of}\;\text{Facility}\;\text{e}\;\\ 0,\;\text{Otherwise}\; \end{array},e=\left\{i,j,w\right\} \end{array}$$

$$\begin{array}{c} OP_{mt}=\{\begin{array}{c} 1,\;\text{Factorym}\;\text{introducesproductiontechnolog}\;t\\ 0,\;\text{otherwise}\; \end{array},m\in M,t\in T \end{array}$$

$$\begin{array}{c} OM_{agb}=\{\begin{array}{c} 1,\text{The}\;\text{product}\;\text{is}\;\text{sent}\;\text{from}\;\text{a}\;\text{to}\;\text{b}\;\text{by}\;\text{means}\;\text{of}\;\text{transportg}\;,\\ 0\text{,}\;\text{otherwise}\; \end{array}a=\left\{m,i,c,j\right\},b=\left\{i,c,j,w,m\right\},g\in G. \end{array}$$

Continuous decision variable

*X*_*agb*_: Indicates the quantity of products shipped from node *a* to *b*, *a* = {*m*, *i*, *c*, *j*}, *b* = {*i*, *c*, *j*, *w*, *m*};

*X*_*mt*_: Represents the number of products produced by factory m using technology *t*.

#### Objective function

Based on the SCLSC network constructed in Fig 1, a multi-objective mathematical pro-gramming model was constructed to minimize network costs, carbon emissions and the largest number of workers.

**Goal 1:** Minimum operation cost of supply chain network (economic benefit).

Eq (1) represents the minimum total cost of supply chain network, including fixed cost of facility establishment, cost of technology introduction to the factory, maintenance cost of transportation vehicle, product production cost, recovery cost, renovation cost, waste disposal cost, transportation cost and cost saved by the production of refurbished parts.

$$\begin{array}{c} \begin{array}{cc} \text{min}\;Z1= & \sum_{e\in \{i,j,w\}}FC_{e}OP_{e}+\sum_{m\in M}\sum_{t\in T}IC_{mt}OT_{mt}\\ & +\sum_{a\in \{m,i,c,j\}b\in \{i,c,j,w,m\}}\sum_{g\in G}[\frac{X_{agb}}{CA_{g}}]GC_{g}OM\prod_{agb}\\ & +\sum_{m\in M}\sum_{t\in T}X_{mt}PC_{mt}+\sum_{c\in C}\sum_{g\in G}\sum_{j\in J}X_{cgj}RC_{j}\\ & +\sum_{j\in J}\sum_{g\in G}\sum_{m\in M}X_{jgm}NC_{j}+\sum_{j\in J}\sum_{g\in G}\sum_{w\in W}X_{jgw}DC_{w}\\ & +\sum_{a\in \{m,i,c,j\}b\in \{i,c,j,w,m\}}\sum_{g\in G}{\left[\frac{X_{agb}}{CAA_{g}}\right]}_{g}DIS_{ab}OMT_{agb}\\ & -\sum_{j\in J}\sum_{g\in G}\sum_{m\in M}X_{jgm}SC_{m} \end{array} \end{array}$$ (1)

**Goal 2:** Most carbon, waste gas, wastewater and solid waste emissions from the network (environ-mental benefits).

Formula (2) represents the minimum total carbon emissions of the supply chain network, in-cluding the carbon emissions generated by the establishment of fixed facilities, the carbon emissions generated by the introduction of technology in the factory, the carbon emissions generated by production, the carbon emissions generated by the renovation of products, the carbon emissions generated by waste disposal and the carbon emissions generated by transportation, waste gas emissions, wastewater emissions and solid waste emissions from products produced in the factory;

$$\begin{array}{c} \begin{array}{cc} \text{min}\;Z2= & \sum_{e\in \{i,j,w\}}EC_{e}OP_{e}+\sum_{m\in M}\sum_{t\in T}IEC_{mt}OT_{mt}+\sum_{m\in M}\sum_{t\in T}X_{mt}PEC_{mt}\\ + & \sum_{j\in J}\sum_{g\in G}\sum_{m\in M}NEC_{j}+\sum_{j\in J}\sum_{g\in G}\sum_{w\in W}X_{jgm}NEC_{j}\\ + & \sum_{j\in J}\sum_{g\in G}\sum_{w\in W}X_{jgw}DEC_{w}\\ + & \sum_{a\in \{m,i,c,j\}}\sum_{b\in \{(i,c,j,w,m\}}\sum_{g\in G}[\frac{X_{agb}}{CA_{g}}]TEC_{g}OM_{ggb}\\ + & \sum_{t\in T}\sum_{e\in \{i,j,w\}}\sum_{n\in V\cap \in N}\sum_{n\in N}DEP_{mn}OP_{e}OP_{mt}\\ + & \sum_{t\in T}\sum_{e\in \{i,j,w\}}\sum_{m\in M}\sum_{n\in N}WEP_{mn}OP_{e}OP_{mt}\\ + & \sum_{t\in T}\sum_{e\{i,i,w\}}\sum_{m\in M}\sum_{n\in N}SEP_{mn}OP_{e}OP_{mt} \end{array} \end{array}$$ (2)

**Goal 3:** The network creates the largest amount of labor (social benefit)

Eq (3) represents the number of jobs created by the network, including the number of jobs created by the establishment of facilities, the number of jobs created by the introduction of technology in factories, the number of jobs needed for production and the number of jobs needed for the maintenance of transportation vehicles.

$$\begin{array}{c} \begin{array}{cc} & \text{min}\;Z3=\sum_{e\in \{i,j,w\}}L_{e}OP_{e}\\ & +\sum_{m\in M}\sum_{t\in T}IL_{mt}OP_{mt}+\sum_{m\in M}\sum_{t\in T}X_{mt}QL_{t}\\ & +\sum_{a\in \{m,i,c,j\}}\sum_{b\in \{i,c,j,w,m\}}\sum_{g\in G}[\frac{X_{agb}}{CA_{g}}]GL_{g}OM_{agb} \end{array} \end{array}$$ (3)

### Constraints

#### Shipping capacity constraints

The transportation constraints determine the capacity limit of the products transported be-tween the centers of each department in the supply chain network. Constraints (4)-(9) ensure that there is product flow between the centers of the relevant departments. Since all depart-ments have production product flows, there are constraints on vehicle transportation between all departments.

Constraint (4) says that shipments to distribution points and markets do not exceed the pro-duction cap of factory M. Constraint (5) means that the distributed shipments do not exceed the total number of distributed products. Constraint (6) states that the transport volume from the market to the recycling station cannot exceed the product inflow to the market. Constraints (7)-(9) state that the production capacity of the recycling center should be greater than or equal to the transportation capacity to the scrap point and to the factory for reprocessing.

$$\begin{array}{c} \sum_{i\in I}X_{mgi}+\sum_{j\in J}X_{jgm}\leq CA_{m},\forall m\in M \end{array}$$ (4)

$$\begin{array}{c} \sum_{c\in C}X_{igc}\leq CA_{i}OP_{i},\forall i\in I \end{array}$$ (5)

$$\begin{array}{c} \sum_{c\in C}X_{cgj}\leq CA_{j}OP_{j},\forall j\in J \end{array}$$ (6)

$$\begin{array}{c} \sum_{c\in C}X_{cgj}\geq CA_{j}OP_{j},\forall j\in J \end{array}$$ (7)

$$\begin{array}{c} \sum_{j\in J}X_{jgw}\geq CA_{w}OP_{w},\forall w\in W \end{array}$$ (8)

$$\begin{array}{c} \sum_{j\in J}x_{jgm}+\sum_{w\leq W}x_{jgw}\leq CA_{j}OP_{j},j\in J \end{array}$$ (9)

#### Network traffic balancing constraints

In order to ensure the flow balance between supply chain network departments, this paper designs the flow constraints as follows. Among them, the constraints (10)-(11) are to ensure that the number of products produced by the factory is equal to the total number of products shipped to the distribution point, and the transportation volume from the factory and from the distribution point is equal. Constraint (12) ensures that the market’s distribution volume to the recycling center is equal to the recycling rate multiplied by the number of products on the market. For the recycling rate, as mentioned in assumption (6), the recycling rate is known. Constraint (13) ensures that the transport volume from the market to the recycling station is equal to the transport volume to the scrap station and refurbishment to the factory. Constraint (14), stating that the recycle site capacity multiplied by the refurbishment rate equals the transport volume from the recycle bin to the factory, and is shown in assumption (6) that the refurbishment rate is known.

$$\begin{array}{c} X_{mt}=\sum_{j\in J}X_{mgj},\forall m\in M,t\in T \end{array}$$ (10)

$$\begin{array}{c} \sum_{m\in M}X_{mgi}=\sum_{c\in C}X_{igc},\forall i\in I \end{array}$$ (11)

$$\begin{array}{c} r_{c}\sum_{i\in I}X_{igc}=\sum_{j\in J}X_{cgj},\forall c\in C \end{array}$$ (12)

$$\begin{array}{c} \sum_{c\in C}X_{cgj}=\sum_{m\in M}X_{jgm}+\sum_{w\in W}X_{jgw},\forall j\in J \end{array}$$ (13)

$$\begin{array}{c} \alpha_{j}\sum_{c\in C}X_{cgj}=\sum_{m\in M}X_{jgm},\forall j\in J \end{array}$$ (14)

#### Demand constraints

Constraint (15), which means that the quantity of products delivered to the market by the distribution point must meet the actual market demand.

$$\begin{array}{c} \sum_{i\in I}X_{igc}\geq D_{c},\forall c\in C \end{array}$$ (15)

#### Factory technology and vehicle constraints

The technical constraint (16) means that each factory can only introduce one technology, so in the corresponding assumption (4), the products produced by the factory are fixed. Constraint (17) indicates that only one vehicle can be selected or not transported, and in hypothesis (7) there are already vehicles of the same type of vehicle.

$$\begin{array}{c} \sum_{t\in T}OP_{mt}\leq 1,\forall m\in M \end{array}$$ (16)

$$\begin{array}{c} \sum_{g\in G}OM_{agb}\leq 1,\forall a=\left\{m,i,c,j\right\},b=\left\{i,c,j,w,m\right\} \end{array}$$ (17)

#### Environmental constraints

Each facility has a fixed carbon footprint, so it doesn’t matter. This paper should consider carbon emissions from factories, transportation vehicles, and various sectors, as well as waste water, waste, and solid emissions for products produced and in manufacturing. Therefore, there are constraints (18)-(19) to express, to ensure that the emissions have numerical values.

$$\begin{array}{c} IEC_{m},PEC_{m},TEC_{g},NEC_{j}DEC_{w}>0 \end{array}$$ (18)

$$\begin{array}{c} DEP_{mn},WEP_{mn},SEP_{mn}>0 \end{array}$$ (19)

#### Decision variable constraints

The following constraints represent the type of variables used by this model. Constraint formulas (19) and (20) reinforce non-negative constraints on decision variables and binary decision variables, respectively.

$$\begin{array}{c} X_{mt},X_{mgi},X_{igc},X_{cgj},X_{jgm},X_{jgw}\geq 0 \end{array}$$ (20)

$$\begin{array}{c} OP_{e},OPT_{mt},OM_{agb}\in \left\{0,1\right\} \end{array}$$ (21)

### Multi-target conversion

**Fig 2:**
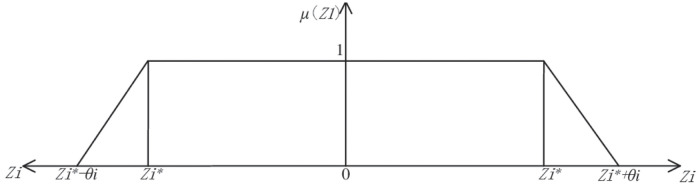
Membership function of each target.

The three objective functions in this paper are optimized and solved. Based on the member-ship degree theory, the membership functions of the three objective functions are established to convert the multi-objective optimization problem into the numerical value of the single objective, that is, the optimization problem to solve the satisfaction degree. The sustainable closed-loop supply chain network model constructed in this paper adopts different member-ship functions for the optimization purposes of the three performance indicators, that is, the number of social employment is required to be as large as possible, and the total supply chain cost and carbon emissions are required to be as small as possible. The membership function images of each target are shown in Fig 2.

As shown in Fig 2, *μ*(*Z*_*i*_)represents the membership degree of the objective function, located at [0, 1]. *Z*_*i*_ represents the value of the objective function, and $Z_{i}^{*}$represents the ideal value of the single optimization objective, namely, the optimal value of the single ob-jective. Θ_*i*_ represents the maximum deviation allowed by the decision maker. The right side of the coordinate axis represents the membership function corresponding to minimizing the target value, while the left side represents the membership function corresponding to max-imizing the target value. Therefore, the membership function of each objective function is shown as follows. Minimize the membership function corresponding to network cost.

$$\begin{array}{c} \mu \left(Z1\right)=\{\begin{array}{cc} 1 & \left(Z1\leq Z1^{*}\right)\\ (Z1^{*}+\theta 1-Z1)/\theta 1, & Z1^{*}\leq Z1\leq Z1^{*}+\theta 1\\ 0 & Z1\geq Z1^{*}+\theta 1 \end{array} \end{array}$$ (22)

Minimize the membership function corresponding to network carbon emissions.

$$\begin{array}{c} \mu \left(Z2\right)=\{\begin{array}{cc} 1 & \left(Z2\leq Z2^{*}\right)\\ (Z2^{*}+\theta 2-Z2)/\theta 2, & Z2^{*}\leq Z2\leq Z2^{*}+\theta 2\\ 0 & Z2\geq Z2^{*}+\theta 2 \end{array} \end{array}$$ (23)

The membership degree function corresponding to the maximum number of networl ment.

$$\begin{array}{c} \mu \left(Z3\right)=\{\begin{array}{cc} 1 & \left(Z3\geq Z3^{*}\right)\\ (Z3-Z3^{*}+\theta 3)/\theta 1, & Z3^{*}-\theta 3\leq Z3\leq Z3^{*}\\ 0 & Z3\leq Z3^{*}-\theta 3 \end{array} \end{array}$$ (24)

$$\begin{array}{c} \max\;\mu \left(\mu \left(Z1\right),\mu \left(Z2\right),\mu \left(Z3\right)\right)=\frac{\mu (Z1)+\mu (Z2)+\mu (Z3)}{3} \end{array}$$ (25)

The single target values were scaled to determine *θ*_1_, *θ*_2_ and *θ*_3_. Due to multi-objective op-timization, the optimized result could not be lower than Zi*, nor exceed the larger values of Zi’and Zi”. Therefore, the value range of *θ*_1_, *θ*_2_ and *θ*_3_ were respectively $0\leq \theta_{1}\leq {Z_{1}}^{*}-\text{min}\{Z_{1}^{\prime },Z_{1}^{\prime \prime }\}$,$0\leq \theta_{2}\leq {Z_{2}}^{*}-\text{min}\{Z_{2}^{\prime },Z_{2}^{\prime \prime }\}$, $0\leq \theta_{3}\leq {Z_{3}}^{*}-\text{min}\{Z_{3}^{\prime },Z_{3}^{\prime \prime }\}$. According to the pref-erence of decision makers, the different degree of expansion can fully reflect their will and preference. Then the multi-objective optimization problem in this paper is transformed into the problem with the greatest satisfaction satisfaction satisfying all the constraints.

## Chaotic particle ant colony algorithm

### PSO algorithm

PSO is based on the observation of bird swarm behavior and the sharing of information by individuals in the flock to realize the evolution process of the whole flock from disorder to order, so as to obtain the optimal solution. Particle swarm has inherent parallelism, intelli-gence of swarm, simple iteration format and fast convergence. The advantage of PSO is that it can easily solve the optimal value of the cooperation or coexistence of multiple particles. The idea of optimization is that the position and speed of each particle are specified. When the particles are affected by their own speed, they also follow the current optimal particle, so as to obtain the optimal value through iteration.

The variable values involved in this process include:

1. The position of the *i*-th particle in the D-dimensional space at time *t* is $X_{i}^{t}=\left(x_{i1}^{t},x_{i2}^{t},\cdots ,x_{iD}^{t}\right)$, that’s a potential solution to the problem.
2. The optimal value of a single particle in the search process, namely the individual optimal solution *p*_*it*_.
3. The optimal value of the particle population in the search process, namely the global optimal solution *p*_*gt*_.
4. In the D-dimensional space, the velocity and position of the particle at time t are respec-tively expressed as $V_{{}_{i}}^{t}=(v_{{}_{i1}}^{t},v_{{}_{i2}}^{t},...v_{{}_{iD}}^{t})$, *P*_*i*_ = (*p*_*i*1_, *p*_*i*2_, …, *p*_*iN*_).

The total particle swarm generates a new generation of population by constantly updating its own velocity and position. The updating formula of particle swarm velocity as follows:

$$\begin{array}{c} V_{id}^{t+1}=\omega V_{id}^{t}+c_{1}r_{1}(p_{id}^{t}-x_{id}^{t})+c_{2}r_{2}(p_{g}^{t}-x_{id}^{t}) \end{array}$$ (26)

The particle position update formula is as follows:

$$\begin{array}{c} x_{{}_{id}}^{t+1}=x_{{}_{id}}^{t}+v_{{}_{id}}^{t+1} \end{array}$$ (27)

In the formula, *ω* represents inertia weight, r1 and r2 represent random numbers between (0, 1), and c1 and c2 represent learning factors. According to the particle velocity updating formula, the velocity of the new generation of particles depends on the influence of particle velocity $\omega V_{{}_{id}}^{t}$, individual memory $c_{1}r_{1}(p_{{}_{id}}^{t}-x_{{}_{id}}^{t})$ and swarm particle $c_{2}r_{2}(p_{g}^{t}-x_{{}_{id}}^{t})$ of the previous generation. The value of inertia weight determines the degree of influence of the velocity of the previous generation on the velocity of the new generation. Based on the above updating method, the particle is constantly approaching the global optimal solution in the later searching process, which is under the dual action of acceleration factor and inertia weight, making the convergence speed and optimization precision of the particle contradict each other. Namely, the higher the accuracy of the algorithm, the slower the convergence rate of the al-gorithm; conversely, the faster the algorithm converges, the easier the particles are to fall into prematurity. In general, although PSO has a relatively good global search ability in the application of solution, the local optimization ability is poor due to the insufficient use of feedback information in the system, and the convergence speed is slow and the phenomenon of stagnation is easy to appear in the later stage. In order to solve the above problems, chaotic ant colony algorithm is used to continue searching in the later stage of the algorithm to avoid the algorithm falling into local optimum.

### Chaotic ant colony algorithm

ACO algorithm is a new parallel heuristic algorithm, which is inspired by the positive feed-back of information between ant colonies in nature and simulates the foraging behavior of ant colonies. This method uses the pheromone content left by ants to identify the optimal degree of the path and has the characteristics of distributed and positive feedback calculation. Let the number of ants in the whole ant colony be *K*, and *n* sites need to be traveled. The distance between site *i* and *j* is *d*_*ij*_(*i*, *j* = 1, 2, …, *n*).

You are free to use colour illustrations for the online version of the proceedings but any print version will be printed in black and white unless special arrangements have been made with the conference organizer. Please check with the conference organiser whether or not this is the case. If the print version will be black and white only, you should check your figure captions carefully and remove any reference to colour in the illustration and text. In addition, some colour figures will degrade or suffer loss of information when converted to black and white, and this should be taken into account when preparing them. The pheromone concentration along paths *i* and *j* at site *t* is $\tau_{ij}^{t}$. The link path between all points is the same at the beginning of the algorithm, namely $\tau_{ij}^{0}=\tau^{0}$. Ant *k*(*k* = 1, 2…*k*) determine the reservoir location to be reached next according to the pheromone concentration of the link path between different points. $P_{ijk}^{t}$ represents the probability of ant *k* moving from reservoiri to reservoir *j* at time *t*.

$$\begin{array}{c} P_{ij}^{k}\left(t\right)=\{\begin{array}{cc} \frac{{\left[\tau_{ij}^{t}\right]}^{\alpha }\cdot {\left[\eta_{ij}^{t}\right]}^{\beta }}{\sum_{s\in A_{k}}{\left[\tau_{ij}^{t}\right]}^{\alpha }\cdot {\left[\eta_{ij}^{t}\right]}^{\beta }}, & s\in A_{k}\\ 0, & s\notin A_{k} \end{array} \end{array}$$ (28)

Where $\eta_{ij}^{t}$ represents the heuristic function, $\eta_{ij}\left(t\right)=\frac{1}{d_{ij}}$, is the probability of the ant going from store *i* to store *j*. *A*_*k*_ = (1, 2, …, *m*) represents the set of places where ant K is to arrive. At the beginning, there are n-1 elements in *A*_*k*_, and when the elements in *A*_*k*_ are empty set, it means that all locations have been reached. *α* is the importance factor of pheromone, and the greater its value is, the greater the role of information concentration in moving is. *β* is the importance factor of heuristic function, and its value is positively correlated with the role of heuristic function in moving process. That is to say, ants prefer to visit sites that are closer than those that are farther away. The pheromone of the connection path between various points volati-lizes with the passage of time. The degree of pheromone volatilization is *ρ*, (0 < *ρ* < 1)so the pheromone concentration on the connection path needs to be updated at any time. The updating mode of pheromone concentration is as follows:

$$\begin{array}{c} \tau_{ij}^{t+1}=\left(1-\rho \right)\tau_{ij}^{t}+\Delta \tau_{ij}^{t} \end{array}$$ (29)

$$\begin{array}{c} \Delta \tau_{ij}\left(t,t+1\right)=\sum_{k=1}^{K}\tau_{ij}^{k}\left(t,t+1\right) \end{array}$$ (30)

$$\begin{array}{c} \tau_{ijk}^{t}=\{\begin{array}{cc} \frac{Q}{L_{k}}, & \;\text{The}\;k_{\text{th}\;}\;\text{ant}\;\text{passes}\;\text{throughthe}\;\left[i,j\right]\;\text{region}\;\\ 0, & \;\text{otherwise}\; \end{array} \end{array}$$ (31)

Where $\Delta \tau_{ij}^{k}$, represents the pheromone concentration released by the *k*_*th*_ at in the paths of *i* and *j*, *Q* is the total pheromone concentration released in the path connecting *i* to *j*, and *L*_*k*_ is the path length.

At the beginning of ACO algorithm, due to the same pheromone concentration in each path, the initial shrinkage blindness and exploratory ability of ant colony are strong. Aiming at the shortcoming of slow convergence speed in the initial stage, chaos system is introduced to improve the pheromone based on the ergodic and randomness of chaos system, and a group of chaotic variables are introduced into Formula (30). Chaotic variables are generated by chaotic mapping, and their expression as follows:

$$\begin{array}{c} R_{ij}^{t}=\lambda R_{ij}^{t}-{\lambda R_{ij}^{t}}^{2} \end{array}$$ (32)

**Fig 3:**
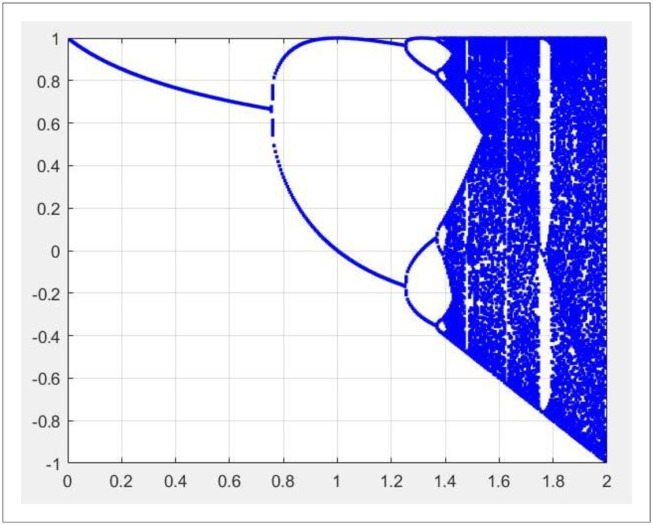
Bifurcation diagram of logistic mapping.

Where, $R_{ij}^{t}$ is chaotic variable; λ is the control variable, and λ is valued at [3.56, 4.0]. When the system is completely chaotic, λ = 4.0, Logistic mapping is used as chaos generator in this paper. When the initial value is $R_{ij}^{0}=0.42212$, the bifurcation diagram of the mapping is shown in Fig 3.

The pheromone updating formula (26) based on chaotic disturbance is further described as:

$$\begin{array}{c} \tau_{ij}^{t+1}=\left(1-\rho \right)\tau_{ij}{}^{t}+\Delta \tau_{ij}{}^{t}+\xi R_{ij}{}^{t} \end{array}$$ (33)

Where, *ξ* is the adjustable coefficient, which is used to adjust the influence of chaos disturbance on pheromone concentration. The introduction of chaos variable makes the pheromone concentration on each path differ in the initial stage, and the ant colony can quickly judge and select the path, thus improving the convergence speed of the algorithm.

## Performance implementation of PSCACO

### Particle coding rules

Firstly, the particle is encoded, and all possible solutions of the multi-objective programming problem are mapped to the search space of the particle swarm optimization algorithm. The decision variables in the paper including binary decision variables are respectively *OP*_*e*_(*e* = {*i*, *j*, *w*}), *OP*_*mt*_, *OM*_*agb*_(*a* = {*m*, *i*, *c*, *j*}, *b* = {*i*, *c*, *j*, *w*, *m*}), Continuous variables are respectively *X*_*ab*_, *X*_*mt*_. Where, the values of *OP*_*e*_, and *OM*_*agb*_ are calculated from the transportation flow of each node, namely, when Xab > 0, OPe = 1 and *OM*_*agb*_ = 1, vice versa, *OP*_*e*_ = 0, *OM*_*agb*_ = 0;*OP*_*mt*_ is derived from the value of *X*_*mt*_, when *X*_*mt*_ > 0, *OP*_*mt*_ = 1; otherwise, *OP*_*mt*_ = 0.

#### Parameter input

Parameters of the optimized model (*FC*_*e*_, *IC*_*mt*_, *PC*_*mt*_*SC*_*m*_, *TC*_*g*_, *RC*_*j*_, *NC*_*j*_, *DC*_*w*_, *GC*_*g*_*EC*_*e*_*IEC*_*mt*_, *PEC*_*mt*_, *TEC*_*g*_, *NEC*_*j*_, *DEC*_*w*_, *DEP*_*mn*_, *WEP*_*mn*_, *SEP*_*mn*_, *L*_*e*_, *IL*_*mt*_, *GL*_*g*_, *QL*_*t*_, *DIS*_*ab*_, *R*_*c*_, *CA*_*m*_, *CA*_*g*_, *CA*_*e*_) for the assignment. Parameters setting of chaotic particle ant colony algorithm: the maximum number of iterations of PSO algorithm *T*1, the maximum number of iterations of ACO algorithm T2, the number of particles and ants K, acceleration factor *c*1, *c*2, initial inertia weight *ω*, initial particle speed and initial particle position.

### Objective function calculation

**Fig 4:**
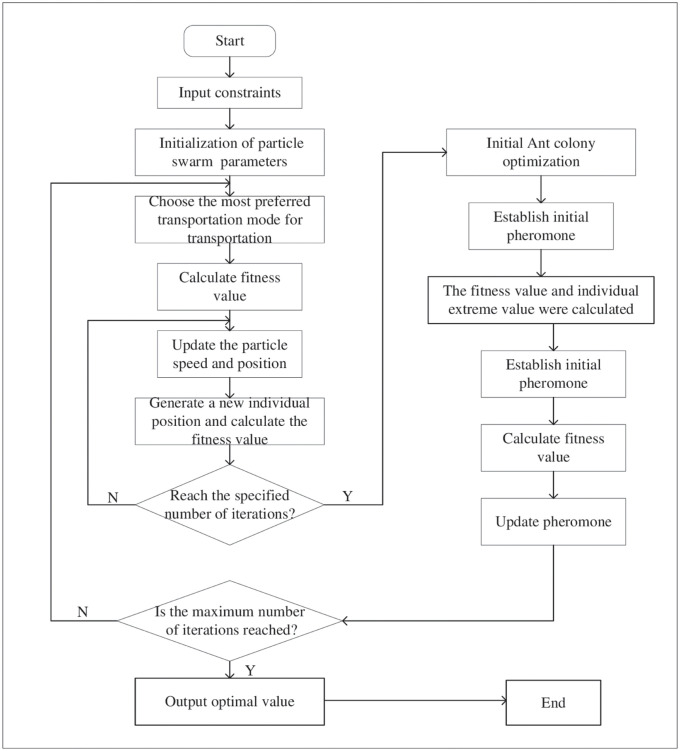
Bifurcation diagram of logistic mapping.

Step1: In order to calculate the product flow between facilities, the constraint (4-21) is encoded into the algorithm language, so as to select the transportation mode with the lowest transportation cost and transportation carbon emission and the largest network labor quantity among facilities.
Step2: In order to meet the demand for products in each market, the material flow ∑_*i*∈*I*_*X*_igc_ is transported to the market from each distribution point under the conditions of constraints (5) and (15); In order to make the quantity of products generated by the factory meet the distribution demand of the distribution point, the material flow ∑_*m*∈*M*_*X*_*mgi*_ is transported from the factory to the distribution point under the conditions of constraints (10) and (11); In order to ensure effective product recycling, the material flow ∑_*c*∈*C*_*X*_*cgj*_ is transported from the market to the recovery point under the conditions of satisfying constraints (6) and (12), while the material flow ∑_*m*∈*M*_*X*_*jgm*_ is transported from the recovery point to the factory under the the condition of traffic volume ∑_*w*∈*W*_*X*_*jgw*_, from the point to waste point. For all the transportation volume mentioned above, prioritize the transportation time, transportation cost and carbon emissions of *G* transportation modes. The smaller the target value, the higher the priority. Select the most preferred mode of transportation until all market needs are met.
Step3: Calculate the fitness value (multi-objective function) under the condition of satisfying the constraint (16-21) *F*(*x*_*i*_) = {min*Z*1(*x*_*i*_), min*Z*2(*x*_*i*_), max*Z*3(*x*_*i*_)} as the fitness value of each initial particle.
Step4: Based on Eqs (26) and (27), the particle velocity and position are updated to generate a new individual position, and the fitness value of the individual at the corresponding position is calculated.
Step5: The fitness values before and after position update were compared, and the current optimal solution was retained to judge the current iteration number t. If *t* < *T*, let *t* = *t* + *l* and go to step 4; If it satisfies *t* > *T*1, the current optimal solution is output.
Step6: The position of the particle obtained by the particle swarm optimization algorithm is taken as the initial solution set of the ant.
Step7: Initialize pheromone concentrations for each path.
Step8: Calculate the fitness value and retain the current optimal solution.
Step9: The pheromone was updated based on Eq (33), and the ant colony transition probability was calculated based on Eq (27). The ant colony position was updated, and the fitness value of the corresponding position was calculated. The fitness value before and after the position update was compared to retain the current optimal solution.
Step10: Determine whether the output condition is satisfied, i.e., *t* > *T*2, then output the current optimal solution; otherwise, let *t* = *t* + *l* and go to Step 8.
Step11: The optimal value of each sub-objective function is calculated, and the solution with the greatest satisfaction in the feasible set is obtained based on Eqs (22) and (25).
According to the above algorithm steps, the algorithm flow chart in Fig 4 can be drawn to clearly see how the improved algorithm operates and optimizes the problem.

### Benchmark experiments and parameters setting

This section is to evaluate the feasibility of the proposed pscaco algorithm, which is solved by using functions with different dimensions. Among them, many scholars also have some research on chaotic ant colony algorithm (CaCO), and verify that CaCO is an effective optimization algorithm. The multi-objective particle swarm optimization algorithm solves the network model and improves the overall systematization. Finally, NSGA-II algorithm is widely used, most of which are multi-objective different problem solving.

This paper compares the optimization results of pscaco algorithm with MOPSO, CaCO and nsga-iiga algorithm to verify the effectiveness, feasibility and running performance of the algorithm. In this paper, MATLAB 2018a programming language, Intel(R)Core(TM) I7-8550CPU, 1.80GHz main frequency, 16GB memory running environment were adopted. The parameters of each algorithm were set as shown in Table 4:

**Table 4:** Algorithm parameter setting.

Algorithm	Parameter values
PSCACO	The maximum number of iterations T1 = 30, T2 = 80, the total number of groups *N* = 100, the inertia weight *ω* = 0.5, the learning factor c1 = 1.5, c2 = 1.3; The importance factorof pheromone *α* = 0.9, the importance factor of heuristic function *β* = 0.1, the total amount of pheromone *A* = 200, and the initial value of chaotic sequence is generated randomly.
CACO	The maximum number of iterations *T* = 100, the number of particles *K* = 100, the importance factor of pheromone *α* = 0.9, the importance factor of heuristic function *β* = 0.1, and the total amount of pheromone *A* = 200.
MOPSO	The maximum number of iterations *T* = 100, the number of particles *N* = 100, the value range of inertia weight *ω* is [0.4, 0.8], the learning factor c1 = 1.5, c2 = 1.3.
NSGA-II	The maximum number of iterations is *T* = 100, the total group size is *K* = 100, the crossover probability is 0.3 and the mutation probability is 0.5

**Fig 5:**
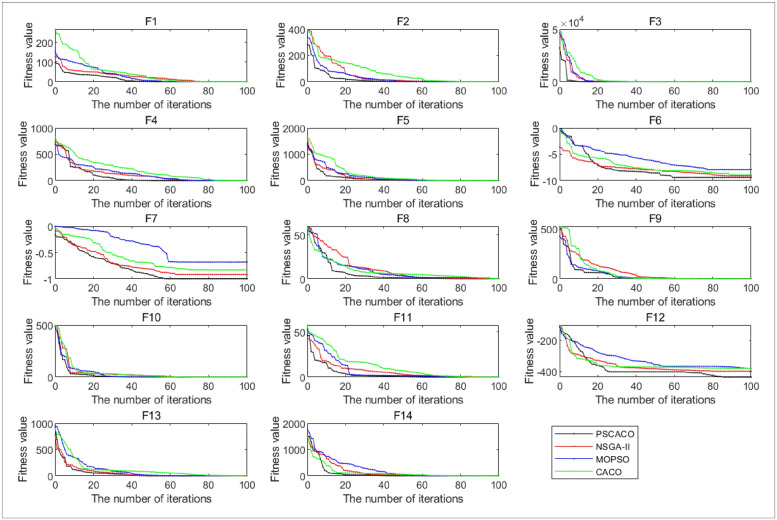
Convergence curve of each test function.

To ensure that the obtained data are comparable, the parameters of each algorithm are set universally, for CACO, MOPSO and NSGA-II algorithms the number of iterations are set to 100, and the total number of iterations for PSCACO is 110. In addition, this section uses 14 test functions for experimentation, and each test function is solved using four algorithms for each execution 10 times, and the optimization Mean and Standard deviation corresponding to each algorithm is obtained as shown in Table 5, where the convergence curve of the optimization result is randomly selected once is shown in Fig 5.

**Table 5:** Algorithm parameter setting.

Test Function Types		PSCACO	NSGA-II	MOPSO	CACO
*f1(Sphere)*	*Mean*	**0.0000E+00**	**0.0000E+00**	1.7666E-51	**0.0000E+00**
	SD	0.0000E+00	0.0000E+00	1.1246E-50	0.0000E+00
*f2(Beale)*	*Mean*	**0.0000E+00**	**0.0000E+00**	**0.0000E+00**	**0.0000E+00**
	SD	0.0000E+00	0.0000E+00	0.0000E+00	0.0000E+00
*f3(Step)*	*Mean*	**0.0000E+00**	**0.0000E+00**	**0.0000E+00**	2.0244E+00
	SD	0.0000E+00	0.0000E+00	0.0000E+00	3.2000E+01
*f4(Griewank)*	*Mean*	**0.0000E+00**	**0.0000E+00**	3.2000E-03	4.7000E-02
	SD	0.0000E+00	0.0000E+00	6.8000E+01	2.4510E+00
*f5(Levy)*	*Mean*	4.4108E-08	1.9822E-02	3.2000E-03	1.3732E-04
	SD	3.500E+06	2.0900E+02	1.3600E+00	5.4320E+00
*f6(Michalewiz)*	*Mean*	-9.8602E+00	-9.0903E+00	-7.0040E+00	-8.9276E+00
	SD	2.0003E-03	1.1000E+01	1.2600E+02	1.4498E+01
*f7(Easom)*	*Mean*	**-1.0000E+00**	-9.1875E-01	-6.8027E-01	**-1.0000E+00**
	SD	0.0000E+00	3.3700E-02	2.0001E+00	0.0000E+00
*f8(Rastrigin)*	*Mean*	5.5030E-07	5.0899E-03	1.6124E-01	3.1217E-02
	SD	1.44000E-03	6.2000E-03	1.3533E+01	2.1000E-04
*f9(Schwefel 1.2)*	*Mean*	1.0000E-026	6.1601E-10	2.3470E-03	**0.0000E+00**
	SD	5.5000E-14	6.2000E-04	4.3700E-02	0.0000E+00
*f10(Schwefel 2.21)*	*Mean*	3.8370E-112	8.9000E-12	8.7050E-04	1.7327E-02
	SD	2.0030E-03	4.0032E-02	7.7023E-02	5.0000E-02
*f11(Schwefel 2.22)*	*Mean*	**0.0000E+00**	1.2000E-05	**0.0000E+00**	8.3000E-08
	SD	0.0000E+00	4.1868E-05	0.0000E+00	4.4489E-04
*f12(Schwefel 2.26)*	*Mean*	-4.1100E+02	-3.9905E+02	-3.8073E+02	-3.8062E+02
	SD	2.4000E+01	1.1866E+02	2.5700E+02	1.5988E+02
*f13(Sum squares)*	*Mean*	**0.0000E+00**	**0.0000E+00**	2.5004E+00	2.3624E+00
	SD	0.0000E+00	0.0000E+00	1.4979E+01	5.2210E-03
*f14(Leon)*	*Mean*	**0.0000E+00**	2.2282E-01	1.3258E-02	**0.0000E+00**
	SD	0.0000E+00	7.9000E-01	7.8800E-01	0.0000E+00

As can be seen in Table 5, the mean and standard deviation of the 10 runs of the test function corresponding to each algorithm, and bold indicates the optimal solution obtained by the algorithm in this model. The results show that from the function f1, only MOPSO does not get the optimal solution in terms of mean and standard variance, while the other three functions get the optimal solution. All four algorithms in function f2 obtain the optimal solution; CACO algorithm in function f3 does not obtain the optimal solution; running algorithms MOPSO and CACO in functions f4 and f13 does not result in an optimal solution, and the other two algorithms result in an optimal solution; the functions f5, f6, f8, f10, f12 do not obtain optimal solutions for the four algorithms run; In function f7, the algorithms PSCACO and CACO yield results in terms of standard deviation this function obtains the optimal solution, for the mean value does not obtain. In f9, the CACO algorithm did not obtain the optimal solution, and the other three algorithms obtained the optimal solution; in f11, both PSCACO and MOPSO obtained the optimal value; f14 obtained the optimal solution in PSCACO and CACO. In f9, the CACO algorithm did not obtain the optimal solution, and the other three algorithms obtained the optimal solution; in f11, both PSCACO and MOPSO obtained the optimal value; f14 obtained the optimal solution in PSCACO and CACO.

In summary, in one randomly selected optimization result, running the PSCACO algorithm are able to obtain in 8 times the optimal solution, which are the functions f1, f2, f3, f14, f7, f11, f13 and f14; The NSGA-II algorithm can obtain the optimal solutions for the test functions f1, f2, f3, f4 and f13, the MOPSO algorithm can obtain the optimal solutions for the test functions f2, f3 and f11, and the CACO algorithm can obtain the optimal solutions for the test functions f1, f2, f7, f9 and f14. In addition, the PSCACO algorithm has better average optimization results for the test functions f5, f6, f8, f10 and In addition, the average optimization results of PSCACO algorithm for test functions f5, f6, f8, f10 and f12 are all better than those of NSGA-II algorithm, MOPSO algorithm and CACO algorithm, and PSCACO algorithm can obtain solutions closer to the global optimum, so the algorithm is feasible.

As can be seen in Fig 5, with approximately the same parameter settings and the same benchmark functions, the trend of the convergence curves of the four algorithms of PSCACO, CACO, MOPSO and NSGA-II obtained from 100 iterations gradually decreases under the gradually increasing number of iterations, slowly decreases at 20 iterations, and basically tends to be constant at 60 iterations. As shown by the convergence curves in Fig 5, the convergence speed and the superiority-seeking ability of the PSCACO algorithm are significantly better than the superior other three algorithms, and it can effectively jump out of the local optimum and is more stable. Based on the above results, it can be seen that the PSCACO algorithm, which integrates the particle swarm algorithm and ant colony algorithm and introduces chaotic perturbation, has a good operational performance because its optimization-seeking accuracy and convergence speed are greatly improved, and it shows its superiority in solving multidimensional test functions.

In the literature [41], different from the traditional ACO-PSO algorithm, the parameters of the two algorithms are fully utilized, and the robust search of particles is relied on to reduce the number of paths. Similarly, the optimization of PID parameters of PSO-ACO in the literature [42] makes the algorithm optimization stable and the running time short. However, they only achieve the improvement of convergence speed, without considering the optimization of the accuracy of the optimization.

## Computational complexity experiment

**Fig 6:**
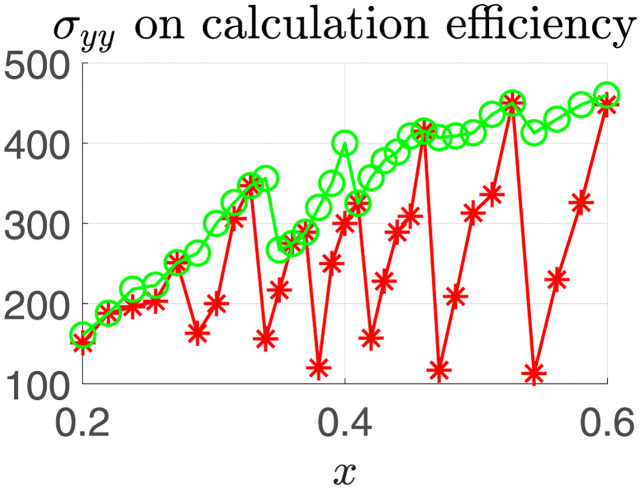
Comparison of calculation efficiency.

In this paper, NURBS curve is used to express the efficiency of the algorithm, so as to reflect the complexity of the algorithm, and one of the multiple benchmark functions is selected for testing. The calculation complexity comparison between the benchmark function algorithm and the algorithm in this paper is shown in Fig 6. In Fig 6, the calculation complexity is expressed by the number of multiplication / division operations required in the calculation process. It can be seen that when the number of curve control points x changes, the computational complexity of the benchmark function method cannot always remain optimal. Although the algorithm in this paper increases a certain amount of computation when making adaptive selection, the computational complexity of the algorithm is only slightly increased compared with the minimum value. Using this algorithm, the computational complexity can be kept at a low level.

In order to further verify the performance of the algorithm in this paper, the above three NURBS curves are used as interpolation tasks to test the time required for interpolation using the benchmark function method and the algorithm in this paper. The weights of the curves are 1 by default. The CPU of all algorithm experimental platforms is Intel (R) core (TM) i7-8550cpu, 1.80GHz dominant frequency, 16GB memory. The experimental results are the average values after multiple runs. The experimental results are shown in the table.

From Table 6, we can see that the average calculation time of the algorithm in this paper is less than that of the benchmark function method, and the calculation efficiency is increased by about 21%. Moreover, with the increase of x value, the improvement of algorithm performance will be more obvious.

**Table 6:** Comparison of calculation time.

NURBS curve	Curve 1	Curve 2	Average time
benchmark function method	15.5 *μ*s	23.7 *μ*s	19.6 *μ*s
the algorithm in this paper	10.3 *μ*s	20.8 *μ*s	15.5 *μ*s

It can be seen that the algorithm performance of the benchmark function method changes relatively with the change of curve x and other parameters when performing NURBS curve interpolation. However, the new algorithm of adaptive selection of interpolation algorithm based on computational complexity overcomes the problem of performance degradation caused by using only one calculation algorithm in traditional methods, and significantly improves the average efficiency of NURBS curve interpolation.

## Discussion

### Numerical examples and simulation

**Fig 7:**
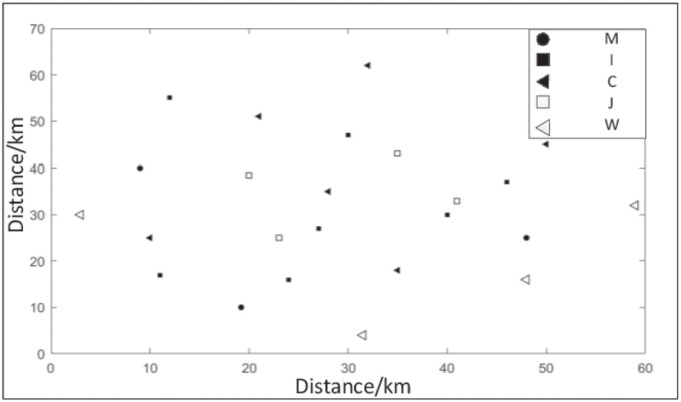
Node location distribution of sustainable closed-loop supply chain network.

To verify the validity of further algorithms and models, a supply chain network of a manufacturing company, which produces, sells and recycles a certain product, is used as a research object. According to the actual situation of the enterprise, it was determined that the manufacturer has 6 customer areas (*c* = 6) and 3 manufacturing plants (*m* = 3) at the location, and the locations of multiple alternative distribution points, multiple alternative recycling points and multiple alternative scrap points were determined, where alternative distribution point *i* = 7, alternative recycling point *j* = 4 and alternative scrap point *w* = 4. The distribution of sustainable closed-loop supply chain network locations is shown in Fig 7.

There are 2 manufacturing technologies available for network introduction (*t* = 2), and the employment coefficients for generating products using different technologies are 0.035 and 0.048, i.e., *QL*_1_ = 0.035 and *QL*_2_ = 0.048, respectively. The values of these parameters are derived by means of a fine-tuning algorithm program. In addition, the remaining network parameters are randomly generated within the uniform distribution interval in combination with the actual operation of the enterprise, as shown in Table 7.

**Table 7:** Relevant parameter value information of transportation means.

Parameter	Vehicle 1	Vehicle 2	Vehicle 3
*GC*_8_	30	30	55
*TC*_*g*_	2.2	2.2	2.7
*CA*_8_	70	70	95
*TEC*_8_	3.6	3.6	5.5
*GL*_8_	0.5	0.5	1

In order to test the effect of optimizing the supply chain network design in three aspects: economic, environmental and social, this section adopts the example values in Section 3.1 and solves the three sub-objective functions of network cost, network environmental pollution emission and network employment quantity by using PSCACO algorithm, NSGA-II algorithm, MOPSO algorithm and CACO algorithm according to the given parameters to obtain the optimal values of each objective function value Z1*, Z2*, Z3*, as shown in Table 8.

**Table 8:** Network parameter setting.

Symbol	Parameters	Symbol	Parameters
*FC*_*i*_	Uniform (8000, 11000)	*DEC*_*w*_	Uniform (0.5, 1.0)
*FC*_*w*_	Uniform (10000, 16000)	*DEP*_*m*1_	Uniform (0.00025, 0.00055)
*FC*_*j*_	Uniform (16000, 19000)	*DEP*_*m*2_	Uniform (0.00015, 0.00035)
*IC*_*m*1_	Uniform (3000, 3500)	*WEP*_*m*1_	Uniform (2.5, 4.5)
*IC*_*m*2_	Uniform (4500, 4700)	*WEP*_*m*2_	Uniform(2.1,3.7)
*PC*_*m*1_	Uniform (4.4.6.4)	*SEP*_*m*1_	Uniform (0.015, 0.035)
*PC*_*m*2_	Uniform (4, 6)	*SEP*_*m*2_	Uniform (0.012, 0.026)
*RC*_*j*_	Uniform (0.4, 0.7)	*α*_*j*_	Uniform (0.65, 0.75)
*NC*_*j*_	Uniform (0.6, 1.1)	*r*_*c*_	Uniform (0.4, 0.64)
*DC*_*w*_	Uniform (0.3, 0.6)	*L*_*i*_	Uniform (2050, 2200)
*D*_*c*_	Uniform (2100, 2300)	*L*_*w*_	Uniform (110, 150)
*EC*_*i*_	Uniform (410, 648)	*L*_*j*_	Uniform (160, 190)
*EC*_*w*_	Uniform (760, 880)	*ILm*1	Uniform (35, 42)
*EC*_*j*_	Uniform (750, 930)	*ILm*2	Uniform (38, 47)
*IEC*_*m*1_	Uniform (36, 41)	*CA*_*m*_	Uniform (4700, 5800)
*IEC*_*m*2_	Uniform (37, 46)	*CA*_*i*_	Uniform (5500, 7900)
*PEC*_*m*1_	Uniform (0.55, 0.70)	*CA*_*w*_	Uniform (2500, 4000)
*PEC*_*m*2_	Uniform (0.45, 0.6)	*CA*_*j*_	Uniform(4000,5000)
*NEC*_*m*_	Uniform (0.8, 1.4)		

**Fig 8:**
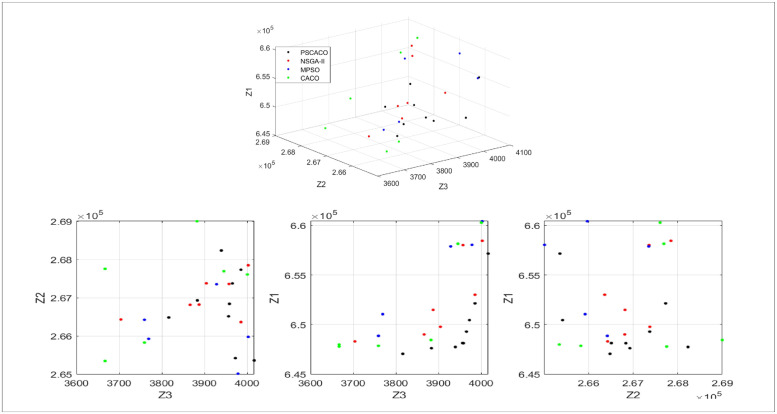
Distribution of feasible solutions with satisfaction degree *μ*> 0.6.

According to the optimization results in Table 7, it can be seen that compared with the NSGA-II algorithm, MOPSO algorithm and CACO algorithm, the use of PSCACO algorithm can obtain the optimal values of each sub-objective function of the sustainable supply chain network, and also shows its superiority in terms of search time. The ideal values of each objective function Z1*, Z2*, and Z3* based on the PSCACO algorithm are obtained as 643806, 262779, and 4124, respectively, and the decision makers take the values 24000, 11000, and 700 for *θ*1, *θ*2 and *θ*3. The distribution of feasible solutions for the overall satisfaction of sustainable supply chain network *μ*>0.6 under each algorithm is shown in Fig 8.

When overall satisfaction *μ* > 0.6, the specific results of PSCACO algorithm for sustainable supply chain network operating costs, network carbon emissions and network employment opportunities are shown in Table 9.

**Table 9:** Multi-objective fuzzy optimization results based on PSCACO algorithm.

*μ*	*μ*_1_	*μ*_2_	*μ*_3_	Z1/yuan	Z2/kg	Z3/people number
0.71	0.47	0.78	0.87	657156	265358	4016
0.69	0.67	0.59	0.83	652136	267731	3985
0.78	0.74	0.78	0.81	650449	265418	3972
0.73	0.78	0.62	0.80	649291	267373	3965
0.76	0.83	0.66	0.79	648120	266839	3958
0.77	0.83	0.69	0.79	648132	266513	3956
0.72	0.85	0.54	0.77	647742	268234	3939
0.73	0.85	0.65	0.70	647614	266929	3883
0.73	0.87	0.69	0.62	647049	266482	3816

As can be seen from Fig 8, when satisfaction *μ* > 0.6, the number of feasible solutions of PSCACO algorithm is Table 9, and the number of feasible solutions satisfying the conditions of NSGA-II algorithm, MOPSO algorithm and CACO algorithm are 7, 5 and 6, respectively. Therefore, the multi-objective model under PSCACO algorithm can obtain more feasible solutions satisfying the satisfaction conditions. In addition, the average satisfaction values of feasible solutions satisfying the conditions of NSGA-II algorithm, MOPSO algorithm and CACO algorithm are respectively: Based on Table 10, it can be seen that the average satisfaction value of the feasible solution satisfying the condition of PSCACO algorithm is 7.36. The optimal satisfaction value *μ* = 0.77. At this time, the operation cost of supply chain network is rmb 648,123, which is rmb 4,226 more than the minimum supply chain network cost under the single goal. The carbon dioxide emission is 266,513 grams, which is 5464 grams more than the minimum supply chain network emission under the single goal, and the network employment opportunities are 3956 people, which is 241 people less than the maximum employment opportunities under the single goal. The above results indicate the effectiveness and superiority of PSCACO algorithm to solve the multi-goal model.

**Table 10:** The optimization results with single objective.

	PSCACO	NSGA-II	MOPSO	CACO
Z1*	643806	644131	644506	644220
Z2*	262839	262994	262879	262779
Z3 *	4124	4119	4074	4115
Elapsed time	9.88 s	12.17 s	12.49 s	22.17 s

In addition, by comparing the membership function and target value of the three targets, it can be seen that the optimal values of multiple objective functions are in conflict with each other, and it is impossible to obtain the optimal values at the same time. However, it can be found that there is a correlation between network cost and network labor quantity. The number of labor created by the network increases with the increase of network cost. This is because the increase in cost is mainly caused by the production capacity, the number of facilities opened and the corresponding production technology changes, and the above network will inevitably create more employment opportunities. There is no obvious correlation between network carbon emissions and network cost and employment opportunities, but its function value changes with the change of the two. In addition, by building the membership function, the decision-maker can set *θ* value according to the enterprise’s own situation, so as to obtain the network planning scheme with the greatest overall satisfaction.

### Sensitivity analysis of model parameters

In this paper, important parameters are selected for sensitivity analysis, so that the model can be optimized and adjusted more finely and accurately. Through parameter optimization, the model can be analyzed and corrected to improve the overall efficiency and accuracy of the network. The important model parameters are selected from the indicators respectively, and the parameters of the same category are integrated. In the case of keeping the other parameters unchanged, the value of each parameter is within the range of [-20%, 20%] and the step size is 5% value change, the objective function value of the model is calculated separately, the selection of important parameters is shown in Table 11, and the calculation results of model parameter sensitivity are shown in Table 12.

**Table 11:** Important parameter selection.

Economics			Environment		Society
Symbol	Parameter Range	Symbol	Parameter Range	Symbol	Parameter Range
*FC*_*i*_	Uniform (6400, 12000)	*PEC*_*m*1_	Uniform (0.44, 0.84)	*L*_*i*_	Uniform(1640,2640)
*IC*_*m*1_	Uniform (2400, 4200)	*NEC*_*m*_	Uniform (0.64, 1.68)	*L*_*j*_	Uniform(128,228)
*PC*_*m*1_	Uniform(3.52,7.68)	*DEC*_*w*_	Uniform (0.4, 1.2)	*ILm*1	Uniform(28,50.4)
*RC*_*j*_	Uniform (0.32, 0.84)	*DEP*_*m*1_	Uniform (0.0002, 0.00066)	*CA*_*m*_	Uniform(3760,6960)
*NC*_*j*_	Uniform (0.48, 1.32)	*WEP*_*m*1_	Uniform (2, 5.4)	*CA*_*i*_	Uniform(4400,9480)
*DC*_*w*_	Uniform (0.24, 0.72)	*SEP*_*m*1_	Uniform (0.012, 0.042)	*CA*_*j*_	Uniform(3200,6000)
*D*_*c*_	Uniform (1680, 2760)	*IEC*_*m*1_	Uniform (28.8, 49.2)		

**Table 12:** Model parameter sensitivity calculation results.

Parameter	-20%	-15%	-10%	-5%	5%	10%	15%	20%
*FC*_*i*_	0.4627	0.4671	0.4705	0.4724	0.479	0.4819	0.4893	0.4948
*IC*_*m*1_	0.4945	0.4917	0.4823	0.4775	0.4735	0.4668	0.4643	0.4623
*PC*_*m*1_	0.5003	0.4981	0.4913	0.4786	0.4732	0.4634	0.4532	0.4501
*RC*_*j*_	0.4963	0.4824	0.4816	0.4787	0.4765	0.4703	0.4693	0.4665
*NC*_*j*_	0.4482	0.4589	0.4620	0.4703	0.4767	0.4969	0.5034	0.5112
*DC*_*w*_	0.4632	0.4687	0.4742.	0.4765	0.4798	0.4801	0.4911	0.4986
*D*_*c*_	0.4991	0.4962	0.4849	0.4806	0.4773	0.4765	0.4688	0.4668
*PEC*_*m*1_	0.4632	0.4697	0.4717	0.4785	0.4835	0.4844	0.4965	0.4997
*NEC*_*m*_	0.4996	0.4968	0.4868	0.4785	0.4776	0.4709	0.4687	0.4650
*DEC*_*w*_	0.4986	0.4924	0.4917	0.4885	0.4735	0.4704	0.4665	0.4597
*DEP*_*m*1_	0.4688	0.4696	0.4785	0.4825	0.4865	0.4908	0.4921	0.4998
*WEP*_*m*1_	0.6578	0.4654	0.4675	0.4684	0.4706	0.4729	0.4858	0.4904
*SEP*_*m*1_	0.4635	0.4685	0.4705	0.4761	0.4896	0.4937	0.4994	0.5007
*IEC*_*m*1_	0.4585	0.4656	0.4785	0.4855	0.4878	0.4902	0.4948	0.5012
*L*_*i*_	0.4988	0.4954	0.4867	0.4785	0.4756	0.4683	0.4604	0.4597
*L*_*j*_	0.4995	0.4927	0.4874	0.4832	0.4789	0.4731	0.4688	0.4643
*IL*_*m*1_	0.4636	0.4697	0.4739	0.4753	0.4801	0.4831	0.4938	0.4993
*CA*_*m*_	0.4603	0.4686	0.4750	0.4795	0.4856	0.4896	0.4947	0.5006
*CA*_*i*_	0.4598	0.4673	0.4698	0.4734	0.4798	0.4824	0.4869	0.4906
*CA*_*j*_	0.4578	0.4678	0.4736	0.4798	0.4865	0.4885	0.4904	0.4978

It can be seen from Table 12 that in the process of calculating the sensitivity of each important parameter, by changing the parameter value range, the value of the objective function is indeed improved or decreased., the sensitivity of environmental and social indicators. After calculation, among the parameters corresponding to economic indicators, the maximum sensitivity is 9.13%, the minimum sensitivity is 2.78%, and the average sensitivity is 5.96%; among the parameters corresponding to environmental indicators, the maximum sensitivity is 4.5%, the minimum sensitivity is 1.58%, The average sensitivity is 3.04%; among the parameters of society, the maximum sensitivity is 5.63%, the minimum sensitivity is 5.45%, and the average sensitivity is 5.54%.

It can be seen that the model parameters under the economic and environmental indicators are more likely to fluctuate to the changes in the value range, and the model is more sensitive to them. The selection of such parameters needs to be more accurate, and the weight of the adjustment according to the experimental results in the simulation is higher.; The change of the parameter sensitivity of society is relatively gentle, but in the process of changing the parameter value of the model, the value of the objective function also has a relatively obvious rise and fall. When adjusting the parameters according to the experimental results, the selection of such parameters needs to be adjusted, but The parameters under the economic cost index of the adjustment weight are slightly lower; the parameter sensitivity under the emission reduction index is lower, and the model is less sensitive to it. The selection range of such parameters does not need to be very precise, and the adjustment weight is the lowest.

## Conclusions

This paper takes sustainable supply chain network as the research object, considers the supply chain network structure with multiple levels and transportation modes for the three factors of sustainable development, and constructs a multi-objective mixed integer planning model for it, aiming to achieve the balance between system economy-environment-society. The mathematical model proposed in this paper and the proposed PSCACO algorithm can efficiently solve the multi-objective sustainable supply chain network design problem and provide decision aids for the sustainability of enterprises.

In view of the advantages and disadvantages of PSO algorithm and ACO algorithm, chaotic variables are introduced and PSCACO algorithm is proposed. The test results of benchmark functions show that PSCACO algorithm can effectively jump out of local optimum and outperforms MOPSO algorithm, CACO algorithm and NSGA-II algorithm in terms of speed of finding the optimum, quality, quantity and accuracy of feasible solutions. In terms of solving the model, the location of the supply chain network nodes is set for the actual situation of the enterprise, and a large number of experiments are conducted using example data simulation arithmetic. The experiments show that in terms of solution quality, the number of feasible solutions of the PSCACO algorithm is nine more than the seven feasible solutions of the MOPSO algorithm, five feasible solutions of the MOPSO algorithm and six feasible solutions of the CACO algorithm, which leads to the feasibility of the PSCACO algorithm for solving the model of this paper.

Based on the affiliation theory, the multi-objective affiliation function is constructed to obtain the overall satisfaction value of the objective function, and the decision-maker is able to obtain a feasible solution that satisfies the condition by giving the allowed deviation range. The simulation results show that the economic benefits are directly proportional to the social benefits, and when the supply chain network cost decreases, the number of network employment opportunities also decreases, while the environmental pollution follows both changes. Therefore, the method can effectively coordinate multi-objective optimization problems of different magnitudes to obtain the network solution with optimal overall satisfaction.

Finally, the sensitivity analysis is carried out on the parameters of the model to calculate the scope of changes in the supply chain benefit evaluation indicators caused by changes in the main variable factors, so that decision makers can comprehensively increase or decrease the weights of changes in the supply chain to reduce and Avoid the influence of unfavorable factors, improve and enhance the effect of supply chain management.

In summary, companies should incorporate sustainability into their supply chain decision making process. Increase strategic collaboration on sustainability between manufacturers, distributors and customers in sustainable supply chains through holistic coordination of economic, environmental and social benefits. It also enables the company to actively participate in environmental pollution issues and social responsibility, so that the development of the company can enter a virtuous cycle.

In addition, for the design of the algorithm, previous researchers have added adaptive adjustment strategies to reasonably adjust the mutation factor and crossover factor to improve the differential evolution algorithm [43]. The neighborhood search algorithm of non-dominated sorting genetic algorithm is used to reduce the delivery cost [44]. Some scholars also designed a parameter adaptive ant colony algorithm PF3SACO based on dynamic hybrid mechanism to accelerate the convergence speed and improve the search ability [45]. Some scholars use mechanisms and strategies to adaptively adjust the search ability of the algorithm. For the algorithm improvement in this paper, it is only improved from the traditional direction, and the adaptive mechanism is not considered. Therefore, all the above methods to improve the algorithm can be studied in depth again, in order to make the optimization effect better.
